# Short form version of the Quality of Trauma Care Patient-Reported Experience Measure (SF QTAC-PREM)

**DOI:** 10.1186/s13104-017-3031-9

**Published:** 2017-12-06

**Authors:** Niklas Bobrovitz, Maria J. Santana, Jamie Boyd, Theresa Kline, John Kortbeek, Sandy Widder, Kevin Martin, Henry T. Stelfox

**Affiliations:** 1 Nuffield Department of Primary Care Health Sciences, University of Oxford, Radcliffe Observatory Quarter, Woodstock Road, Oxford, OX2 6GG UK; 2 Department of Community Health Sciences, University of Calgary, Calgary, AB Canada; 3 Department of Psychology, University of Calgary, Calgary, AB Canada; 4 Department of Surgery, University of Calgary, Calgary, AB Canada; 5 Department of Surgery, University of Alberta, Edmonton, Canada; 6 Department of Critical Care Medicine, University of Alberta, Edmonton, Canada; 7 Chinook Regional Hospital, Lethbridge, Canada; 8 Department of Critical Care Medicine, University of Calgary, Calgary, AB Canada; 9 Department of Medicine, University of Calgary, Calgary, AB Canada

**Keywords:** Patient experience, Patient reported measure, Injury, Trauma, Quality of care

## Abstract

**Objective:**

To enable the valid and reliable measurement of patient experiences we previously published a multicenter multi-center validation of the Quality of Trauma Care Patient-Reported Experience Measure (QTAC-PREM). The purpose of this study was to derive a simplified, short form version of the QTAC-PREM to further enhance the feasibility of measuring patient experiences in injury care. To identify candidate items for the short form we reviewed the results of the original multi-center long form validation cohort study, which included 400 injury care patients and their family members recruited from three trauma centers. We only included the best performing items on the revised short form.

**Results:**

The acute care component of the measure was shortened by 30% and the post-acute care component was shortened by 42%. We identified two subscales on the acute measure *(information and communication; clinical and ancillary care*) and one subscale on the post-acute measure (*post*-*discharge information and communication*). The measurement properties of the short form measure were similar to that of the validated long form. This short form assessment of patient injury care experiences offers a useful, practical, and easy tool for trauma centers to implement for service evaluation, quality improvement, and injury care research.

**Electronic supplementary material:**

The online version of this article (10.1186/s13104-017-3031-9) contains supplementary material, which is available to authorized users.

## Introduction

Governments, health care regulators, funders, and researchers have made clear that the processes and outcomes of care should align with patients’ needs, preferences, and values [1]. Therefore, patient experience is a central component of the quality of care and assessing it is necessary to evaluate and improve care.

The value of measuring patient experiences has been demonstrated. For example, patient experiences have been used to identify gaps in care and guide quality improvement in primary care [2–4] and medical, surgical, and obstetrical inpatient care [5, 6]. To date, there have been few studies of patient experiences in injury care; however, there is evidence to suggest that injury care experiences could be utilized to inform health service delivery. For example, researchers in the Victorian State Trauma System conducted interviews with patients and identified specific, actionable targets for improvement based on patients’ perceived gaps in care [7]. This study is valuable for informing local service design however, validated measurement tools are needed to enable trauma programs to efficiently and routinely incorporate patient experiences into quality measurement and improvement.

To enable the valid and reliable measurement of patient experiences we previously developed [8] and validated [10] the Quality of Trauma Care Patient-Reported Experience Measure (QTAC-PREM). This was the first measure of injury care experiences to capture both the acute and post-acute phases of care. We found that the measure was feasible to use and had evidence of validity and reliability. However, the tool examines 63 items across five domains and can take considerable time to complete. This may act as a barrier to utilization in routine practice [11, 12].

The purpose of this analysis was therefore to derive a simplified, short form version of the QTAC-PREM to further enhance the feasibility of measuring patient experiences in injury care. In this article we report the derivation and key measurement properties of the new SF QTAC-PREM.

## Main text

### Methods

#### Description of the long form QTAC-PREM and validation study

The long form QTAC-PREM is a patient experience measure consisting of two parts administered separately: a 35-item acute care component to be self-completed in-hospital by patients (or patient proxy) and a 28-item post-acute care component administered via telephone interview 2–3 months after hospital discharge. The measure includes both close-ended and open-ended items. The multi-step development of the original scale included a literature review, focus groups with patients and providers, prospective single-center pilot-testing with 134 participants [8], revision using cognitive testing [9], and a prospective multi-center validation with 400 participants [10].

#### Data sources and content flagged for deletion

We used the quantitative and qualitative data from our multi-center validation study to inform our selection of items for the short form [10].

We flagged for deletion items: (1) that did not form part of existing subscales (2) to which 20% or more of patients selected “not-applicable” or “not able to answer” response options, (3) that did not correlate highly with the overall rating of the quality of care, (4) were shown to be redundant by measures of collinearity, (5) that were conceptually redundant, (6) with the lowest test–retest reliability coefficients, and (7) with limited response variation.

We also used the qualitative responses that participants provided to open-ended questions and documented participants’ comments obtained during administration of the post-acute measure via telephone interview.

#### Dataset and statistical analysis

To assess the operating characteristics of the new combination of items appearing on the short form we repeated the analyses using data from the original multi-center prospective cohort study [10]. The dataset included acute care measure responses from 400 participants recruited between June 2012 and November 2013 from three trauma centers in Canada: two Level 1 trauma centers (University Hospital, Edmonton, and Foothills Medical Center, Calgary) and one Level 3 trauma center (Chinook Regional Hospital, Lethbridge). Follow-up post-acute care interviews were completed on 207 of the 400 participants.

We conducted factor analysis to identify subscales; assessed the internal consistency of the subscales using Cronbach’s alphas; and assessed construct validity by calculating univariate (Spearman correlations) and multivariate (ordinal regression) associations between the subscales and global rating item to determine if the subscales were predictors of overall ratings of the quality of care.

We used only complete cases for all analyses (i.e. cases with missing data or ‘not applicable’ or ‘not able to answer’ response selections were excluded).

We have re-reported the test–retest reliability coefficients for the retained items and spearman correlations between individual items and the global rating item obtained during the long form validation. These properties are expected to remain stable between the long form and short form versions of the measure.

### Results

The acute care measure was shortened to 24 items and the post-acute measure was shortened to 16 items. The final short form versions of the measure are available in the Additional files 1, 2, 3, 4.

To derive the short form the following changes were made: 19 items were eliminated; 33 items were retained with no revision or minor revision to wording or response options; 2 items assessing general health status were replaced by 4 validated items assessing physical and mental health status separately; and 2 items assessing the provision and adequacy of discharge information were merged to create a single item. Detailed explanations of the item selections and revisions for both short form components can be found in Additional files 5, 6.

#### Identification of subscales

The acute care factor analysis included 154 complete cases (Table 1). We identified a two-factor solution accounting for 74% of observed variance. Factor 1, *information and communication,* included items on the scope, clarity, consistency, and availability of acute care information and communication. Factor 2, *clinical and ancillary care*, included items assessing the clinical processes of care (e.g., pain well-controlled) and ancillary components including being treated unfairly. Spearman’s correlations between the subscales were moderate (0.48) suggesting that the subscale scores should not be combined to form an overall acute care score.

**Table 1:** Measurement properties of the short form QTAC-PREM acute care measure

Subscales Item numbers and descriptions^a^	Sub-scale factor loadings (n = 154)^b^	Subscale Cronbach’s alpha and corrected item-subscale correlations, (n = 400)^c^	Item/subscale to global rating item correlations^d^, (n = 400)^c^	Reliability coefficient (95% CI), (n = 78)^e^
Information and communication	–	0.55	0.50^g^	
1. All injuries explained (10)	0.83	0.50	0.34^g^	0.51 (0.13–0.88)^h^
2. Explained self-care (12)	0.75	0.43	0.29^g^	0.64 (0.41–0.86)^h^
3. Explained recovery timeline (13)	0.96	0.43	0.19^g^	0.69 (0.45–0.94)^h^
4. Consistent information (14)	0.35	0.50	0.47^g^	0.78 (0.68–0.85)
Clinical and ancillary care	–	0.85	0.67^g^	
5. Pain well-controlled (18)	0.68	0.84	0.50^g^	0.72 (0.59–0.81)
6. Helped with agitation (21)	0.75	0.81	0.57^g^	0.71 (0.55–0.82)
7. Handled carefully (22)	0.83	0.83	0.44^g^	0.64 (0.48–0.76)
8. Helped with hygiene (23)	0.60	0.84	0.40^g^	0.68 (0.52–0.79)
9. Providers explained their roles (24)	0.61	0.83	0.44^g^	0.78 (0.67–0.85)
10. Addressed concerns (25)	0.70	0.82	0.55^g^	0.68 (0.52–0.80)
11. Dignity considered (27)	0.77	0.82	0.44^g^	0.57 (0.39–0.70)
12. Offered to discuss emotional needs (26)^f^	–	–	–	–
13. Perceived safety of care (28)	0.76	0.85	0.34^g^	0.88 (0.82–0.92)
14. Treated unfairly (30)	0.80	0.85	0.28^g^	0.44 (0.24–0.61)
Stand-alone items	–			
15. Global rating (34)	–	–		0.85 (0.77–0.90)

^a^Corresponding long form item number in (brackets)

^b^n = 154 complete cases available out of total sample of 400 completed surveys

^c^Sample size varied depending on the number of complete cases in each sub-scale: *clinical and ancillary care* (n = 225) and *information and communication* (n = 273)

^d^Spearman’s correlations, including Bonferroni correction to account for multiple testing

^e^Intraclass correlation coefficient unless otherwise indicated

^f^This item underwent major revision for inclusion on the short form and, therefore, could not be assessed using data from the long form validation study. However, we have included it within the *clinical and ancillary care* subscale as it conceptually fits within this construct

^g^Significant at the p < 0.01 level

^h^Cohen’s Kappa coefficient

The post-acute care factor analysis included 117 complete cases (Table 2). A one-factor solution accounting for 78% of observed variance was identified. The factor, *post*-*discharge information and communication*, included items on the provision, adequacy, scope, and availability of follow-up information and communication.

**Table 2:** Measurement properties of the short form QTAC-PREM post-acute care measure

Subscales Item numbers and descriptions^a^	Sub-scale factor loadings (n = 117)^b^	Subscale Cronbach’s alpha and corrected item-subscale correlations, (n = 117)^b^	Subscale to global rating item correlations^d^, (n = 207)^c^	Reliability coefficient (95% CI), (n = 76)^e^
Post-discharge information and communication		0.64	0.61^g^	
1. Received adequate written instructions (2/3)	0.40	0.27	0.25^g^	0.78 (0.67–0.86)
7. Explained next steps in recovery (9)	0.82	0.58	0.52^g^	0.71 (0.52–0.91)^h^
8. Described recovery timeline (10)	0.55	0.34	0.29^g^	0.55 (0.35–0.76)^h^
9. Understandable explanations (12)	0.74	0.57	0.43^g^	0.69 (0.55–0.80)
12. Post-discharge recovery guidance (17)	0.68	0.55	0.79^g^	0.86 (0.79–0.91)
Stand-alone items				
2. Pain management (4)^f^	–	–	0.25^g^	0.90 (0.77–1.00)^h^
5. Difficulty scheduling appointments (8a–c)^i^	–	–	–	–
10. Family physician informed (16)^f^	–	–	0.40^g^	0.87 (0.78–0.92)
11. Perceived safety of care (15)^j^	–	–	0.10	0^k^
13. Global rating item (18)	–	–	–	0.90 (0.83–0.94)

^a^Corresponding long form item number in (brackets)

^b^n = 117 complete cases available out of total sample of 207 completed surveys

^c^The available sample size varied from n = 118 to n = 207 depending on the number of complete cases for the subscale/individual items

^d^Spearman’s correlations

^e^Intraclass correlation coefficient unless otherwise indicated

^f^Item did not load onto a sub-scale

^g^Significant at the p < 0.01 level

^h^Cohen’s Kappa coefficient

^i^This item underwent major revision for inclusion on the short form and therefore could not be assessed using data from the long form validation study. However, we recommend it be reported as a stand-alone item as it does not conceptually match the construct of the subscale

^j^Item excluded from factor analysis due to limited variation among complete cases

^k^No reliability estimate because of limited response variance among the sample of test–retest respondents

#### Internal consistency

Subscale Cronbach’s Alpha values were 0.55 and 0.87 for the acute measure subscales (Table 1) and 0.64 for the post-acute care subscale (Table 2). Corrected item—subscale correlations ranged from 0.43 to 0.85 for the acute care measure (Table 1) and from 0.18 to 0.64 for the post-acute measure (Table 2).

#### Construct validity

All subscales and items on the acute care measure had significant univariate correlations (p < 0.01) with the global rating item (Table 1). In multivariate analysis (n = 154), *information and communication* (p < 0.001) and *clinical and ancillary care* (p < 0.001) were independently associated with the global rating item (Additional file 7).

Univariate analysis of the post-acute measure showed that the subscale and eight of nine items were significantly associated with the global rating item (Table 2). In multivariate analysis (n = 117), the subscale (p < 0.001) and one of the stand-alone items (item 10, *family physician informed*, p = 0.01) were independently associated with the global rating item, while the item on *pain management* (Item 4, p = 0.09) and *perceived safety of care* (Item 11, p = 0.57) were not (Additional file 7).

#### Revisions to simplify analysis and reporting

The response options “not applicable” and “not able to answer” introduce challenges in the analysis and interpretation of results [1]. We identified these challenges in our validation study and they have been noted in other survey development efforts including the Consumer Assessment of Health Plans [13]. Therefore, we eliminated these options from the short form patient versions of the tools. A version of these response options was retained for the proxy-respondent tools, but we replaced “not applicable” and “not able to answer” with “I don’t know” as this option aligned more closely with proxy participants’ language during telephone interviews in the validation study.

#### Other revisions

We changed the phrase “healthcare providers” to “healthcare practitioners”. In some health systems, such as the United States, “healthcare providers” may be interpreted to include payers that finance or reimburse the cost of health services. However, our intention is to ask about practitioners that deliver care.

### Discussion

These analyses were conducted to derive short form versions of the Quality of Trauma Care Patient-Reported Experience Measure (QTAC-PREM). We shortened the measure by 23 items. The operating characteristics of the short form QTAC-PREM identified in this study are comparable with other validated and widely used measures of patient and family experiences of care [14–16].

We were able to identify subscales on the short form that will allow for simplified reporting and interpretation. The acute care short form consists of two subscales (*clinical and ancillary care, information and communication*), while the post-acute care measure consists of one (*post*-*discharge information and communication).*

The subscales on both short form measures showed adequate internal consistency, indicating that summary subscale scores would accurately represent item level data [17, 18]. We also found evidence of construct validity, as all the subscales were significant, independent predictors of patients’ overall ratings of care.

The measurement properties of the short form measure are similar to those of the validated long form. The only notable differences include slightly lower internal consistency of the short form acute care subscale *information and communication* compared to the long form information-related subscales (0.55 vs. 0.67 and 0.76). This may be the result of merging items from two scales on the long form that assessed narrower constructs to form a single scale addressing a more generic construct; internal consistency is lower when the construct measured is more generic [17]. The scale also has few items, which is known to affect internal consistency. This value suggests that a summary score for the scale is not a perfect representation of the underlying item scores and is a trade-off to gain the benefits of a shorter, more efficient instrument.

There are three benefits of using the short form QTAC-PREM as opposed to the long form. First, response burden will be reduced. Second, the short form may be a more efficient way to obtain informative and comparative data on quality of care. We slightly revised item wording and response options such that the content of the short form should be applicable to all injury patients that are hospitalized and discharged alive. As a result, this should increase the amount of useable and comparable data from each administered measure. Third, the results can be summarized and reported more efficiently. A greater proportion of items fit within subscales compared to the long form and therefore, the results can be summarized with fewer metrics.

The QTAC-PREM and SF QTAC-PREM provide two complementary instruments for assessing patient experiences. The QTAC-PREM may be more relevant for research or detailed audits given its larger number of items, while the short-form may be suited for more frequent applications, such as routine audits. We believe both instruments are potentially important tools for measuring care and guiding quality improvement initiatives.

### Conclusion

Assessing patient experience is vital for designing and delivering high-quality injury care. To increase the feasibility of measuring patient experiences we derived a short form version of the Quality of Trauma Care Patient-Reported Experience Measure (SF QTAC-PREM). The short form tool has evidence of validity and reliability. The SF QTAC-PREM offers a useful, practical, and easy tool for trauma centers to implement for service evaluation, quality improvement, and injury care research.

## Limitations

This study has some limitations. First, the data used in this study were obtained using the long form measure and, therefore, the characteristics of the items may vary slightly when implemented as a short form. Although this method has been utilized to derive and validate widely-used and highly cited scales, such as the short form oral health impact profile [19] and the stroke impact scale [20], future research should verify the measurement properties by administering the short form version to another population. Second, we made minor revisions to the wording and response options of some of the items retained for the short form and this may affect their measurement properties. Therefore, verifying the operating characteristics may be of value. However, the revisions have simplified the tool for end-users and have been informed by evidence. Therefore, it is likely that the properties of the items that may require re-validation will be acceptable.

## Additional files

**Additional file 1.** Quality of Trauma Care Patient-Reported Experience Measure (QTAC-PREM)—Short Form. Part 1: Acute Care, Patient Survey.

**Additional file 2.** Quality of Trauma Care Patient-Reported Experience Measure (QTAC-PREM)—Short Form. Part 1: Acute Care, Family Member/Proxy Survey.

**Additional file 3.** Quality of Trauma Care Patient-Reported Experience Measure (QTAC-PREM)—Short Form. Part 2: Post-Acute Care, Patient Survey.

**Additional file 4.** Quality of Trauma Care Patient-Reported Experience Measure (QTAC-PREM)—Short Form. Part 2: Post-Acute Care, Family Member/Proxy Survey.

**Additional file 5.** Mapping the derivation of the short form acute care QTAC-PREM from the original long form items.

**Additional file 6.** Mapping the derivation of the short form post-acute care QTAC-PREM from the original long form items.

**Additional file 7.** Short form acute care QTAC-PREM ordinal logistic regression results, Short form post-acute care QTAC-PREM ordinal logistic regression results.

## Abbreviations

SF QTAC-PREM: short form Quality of Trauma Care Patient Reported Experience Measure
QTAC-PREM: Quality of Trauma Care Patient Reported Experience Measure
