# Supplementary material for: Short form version of the Quality of Trauma Care Patient-Reported Experience Measure (SF QTAC-PREM)
Source: BMC Res Notes. 2017 Dec 6;10:693. doi: 10.1186/s13104-017-3031-9 (PMC5718023; doi:10.1186/s13104-017-3031-9)
Supplement: Supplementary file 1 — Additional file 1. Quality of Trauma Care Patient-Reported Experience Measure (QTAC-PREM)—Short Form. Part 1: Acute Care, Patient Survey. [file 13104_2017_3031_MOESM1_ESM.docx]

**Quality of Trauma Care Patient-Reported Experience Measure (QTAC-PREM) - Short Form**

**Part 1: Acute Care, Patient Survey**

| ***Read*** |
| --- |
| - This survey contains questions about your experiences of care. - Your care for this injury may have included: care in the emergency department, intensive care unit, trauma care unit, and rehabilitation unit in the hospital. - Think ONLY about the care you have received for this injury when answering the questions. - Please CHECK ONLY ONE response for each question. - All information is confidential (your healthcare practitioners will not see this information). |

| ***During your care for this injury…*** | | |
| --- | --- | --- |
|  | | |
| **1.** | | **Did your healthcare practitioners clearly explain all your injuries to you in a way**  **you could understand?** |
|  | No | |
|  | Yes, but I wanted more information | |
|  | Yes and I got all the information I wanted | |
|  |  | |
| **2.** | **Did your healthcare practitioners give instructions on how you should care for your injuries?** | |
|  | No | |
|  | Yes, but I wanted more information | |
|  | Yes and I got all the information I wanted | |
|  |  | |
| **3.** | **Did your healthcare practitioners discuss how long it might take you to recover from your injuries?** | |
|  | No | |
|  | Yes, but I wanted more information | |
|  | Yes and I got all the information I wanted | |
|  |  | |
| **4.** | **How often was the information you received from your various healthcare practitioners consistent?** | |
|  | Never | |
|  | Sometimes | |
|  | Usually | |
|  | Always | |
|  |  | |
| **5.** | **How often was your pain well controlled?** | |
|  | Never | |
|  | Sometimes | |
|  | Usually | |
|  | Always | |
|  |  | |
| **6.** | **How often did the healthcare practitioners**  **do everything they could to help you with your discomfort, agitation or irritability?** | |
|  | Never | |
|  | Sometimes | |
|  | Usually | |
|  | Always | |
|  |  | |

| **8.** | **How often did the** **hospital staff offer to help you maintain your personal hygiene?** |
| --- | --- |
|  | Never |
|  | Sometimes |
|  | Usually |
|  | Always |
|  |  |
| **9.** | **When meeting a new healthcare practitioner for the first time how often did they introduce themselves and clearly explain their role in your care?** |
|  | Never |
|  | Sometimes |
|  | Usually |
|  | Always |
|  |  |
| **10.** | **When you had questions, concerns, or frustrations about your care how often did your healthcare practitioners take action?** |
|  | Never |
|  | Sometimes |
|  | Usually |
|  | Always |
|  |  |
| **11.** | **How often did your healthcare practitioners treat you with dignity?** |
|  | Never |
|  | Sometimes |
|  | Usually |
|  | Always |
|  |  |
| **12.** | **Did a healthcare practitioner (e.g. nurse, social worker, psychologist) offer to speak with you about your mental or emotional health?** |
|  | No, but I did not need support |
|  | No and I felt I needed support |
|  | Yes, but I did not need support |
|  | Yes, but I needed more support |
|  | Yes and I got all the support I needed |
|  |  |
| **13.** | **How often did you experience care that was unsafe?** |
|  | Never |
|  | Sometimes |
|  | Usually |
|  | Always |
|  |  |
| **14.** | **How often were you treated unfairly because of your age, ethnicity, gender, cultural beliefs, religious beliefs, or other personal characteristics?** |
|  | Never |
|  | Sometimes |
|  | Usually |
|  | Always |

| **7.** | **When the healthcare practitioners helped you to move around (i.e., change position in bed, walking etc.) how often did they do it carefully?** |
| --- | --- |
|  | Never |
|  | Sometimes |
|  | Usually |
|  | Always |
|  |  |

| ***Overall Care*** | |
| --- | --- |
|  | |
| **15.** | **Please provide an overall rating of the care**  **you have received for this injury.** |
|  | 0 - Worst Injury Care Possible |
|  | 1 |
|  | 2 |
|  | 3 |
|  | 4 |
|  | 5 |
|  | 6 |
|  | 7 |
|  | 8 |
|  | 9 |
|  | 10 - Best Injury Care Possible |
|  |  |
| **16.** | **Since being injured, which of the following options best describes your current overall physical health?** |
|  | Excellent |
|  | Very good |
|  | Good |
|  | Fair |
|  | Poor |
|  |  |
| **17.** | **Since being injured, which of the following options best describes your current overall mental or emotional health?** |
|  | Excellent |
|  | Very good |
|  | Good |
|  | Fair |
|  | Poor |
|  |  |
| **18.** | **Please provide comments on how we can**  **improve injury care for patients.** |
|  | |
|  | |
|  | |
|  | |
|  | |
|  | |
|  | |
|  | |
|  | |
|  | |

| ***Questions about you*** | | | | | |
| --- | --- | --- | --- | --- | --- |
|  | | | | | |
| **19.** | **I am...** | | | | |
|  | Male | | | | |
|  | Female | | | | |
|  |  | | | | |
| **20.** | **What is your age? (e.g., 40 years old)** | | | | |
|  | | | | | |
|  | | | | | |
| **21.** | **How were you injured?** | | | | |
|  | Car crash | | | | |
|  | ATV/off-roading vehicle crash | | | | |
|  | Pedestrian/bicycle hit by a motor vehicle | | | | |
|  | Bicycle crash | | | | |
|  | Fall | | | | |
|  | Assault | | | | |
|  | Burn | | | | |
|  | Self-harm | | | | |
|  | Other (please specify): | | |  | |
|  | | | | | |
| **22.** | | | **What language do you mainly speak at home?** | | |
|  | | | English | | |
|  | | | French | | |
|  | | | Other (please specify): | |  |
|  | | | | | |
| **23.** | | **What is the highest level of education that you have completed?** | | | |
|  | | 8th grade or less | | | |
|  | | Some high school, but did not graduate | | | |
|  | | High school or high school equivalency | | | |
|  | | Some college/university, did not graduate | | | |
|  | | College, CGEP, or other non-university certificate or diploma | | | |
|  | | University degree | | | |
|  | | Post-graduate degree or professional designation | | | |
|  | |  | | | |
| **24.** | **Do you consider yourself to be…** | | | | |
|  | White | | | | |
|  | Chinese | | | | |
|  | First Nations, Metis, Inuk, Aboriginal, or Indigenous | | | | |
|  | South Asian (East Indian, Pakistani, Sri Lankan, etc.) | | | | |
|  | Black | | | | |
|  | Other (please specify): | | |  | |
