# Supplementary material for: Short form version of the Quality of Trauma Care Patient-Reported Experience Measure (SF QTAC-PREM)
Source: BMC Res Notes. 2017 Dec 6;10:693. doi: 10.1186/s13104-017-3031-9 (PMC5718023; doi:10.1186/s13104-017-3031-9)
Supplement: Supplementary file 2 — Additional file 2. Quality of Trauma Care Patient-Reported Experience Measure (QTAC-PREM)—Short Form. Part 1: Acute Care, Family Member/Proxy Survey. [file 13104_2017_3031_MOESM2_ESM.docx]

**Quality of Trauma Care Patient-Reported Experience Measure (QTAC-PREM) – Short Form**

**Part 1: Acute Care, Family Member/Proxy Survey**

| ***Read*** |
| --- |
| - This survey contains questions about your family member's experiences of care. - When completing this survey remember that you are completing it for the patient. - Think about how the patient would answer the questions. - The patient’s care for this injury may have included: care in the emergency department, intensive care unit, trauma care unit, and rehabilitation in the hospital. - Think ONLY about the care the patient has received for this injury when answering the questions. - Please CHECK ONLY ONE response for each question. - All information is confidential (the healthcare practitioners will not see this information). |

| **7.** | **When healthcare practitioners helped the patient to move around (i.e., change position**  **in bed, walking etc.) how often did they do it**  **carefully?** |
| --- | --- |
|  | Never |
|  | Sometimes |
|  | Usually |
|  | Always |
|  | I don’t know |
|  |  |
| **8.** | **How often did the hospital staff offer to help maintain the patient’s personal hygiene?** |
|  | Never |
|  | Sometimes |
|  | Usually |
|  | Always |
|  | I don’t know |
|  |  |
| **9.** | **When meeting a new healthcare practitioner for the first time how often did they introduce themselves and explain their role in the patient’s care?** |
|  | Never |
|  | Sometimes |
|  | Usually |
|  | Always |
|  | I don’t know |
|  |  |
| **10.** | **When you or the patient had questions, concerns, or frustrations about their care, how often did** **the healthcare practitioners take action?** |
|  | Never |
|  | Sometimes |
|  | Usually |
|  | Always |
|  | I don’t know |
|  |  |
| **11.** | **How often did the healthcare practitioners treat the patient with dignity?** |
|  | Never |
|  | Sometimes |
|  | Usually |
|  | Always |
|  | I don’t know |
|  |  |

| ***During your family member’s care for this injury…*** | |
| --- | --- |
|  | |
| **1.** | **Did the healthcare practitioners clearly explain all of the patient’s injuries in a way you could understand?** |
|  | No |
|  | Yes, but I wanted more information |
|  | Yes and I got all the information I wanted |
|  | I don’t know |
|  |  |
| **2.** | **Did the healthcare practitioners give instructions on how to care for the patient’s injuries?** |
|  | No |
|  | Yes, but I wanted more information |
|  | Yes and I got all the information I wanted |
|  | I don’t know |
|  |  |
| **3.** | **Did the healthcare practitioners discuss how long it might take the patient to recover from their injuries?** |
|  | No |
|  | Yes, but I wanted more information |
|  | Yes and I got all the information I wanted |
|  | I don’t know |
|  |  |
| **4.** | **How often was the information that was given by the various healthcare practitioners consistent?** |
|  | Never |
|  | Sometimes |
|  | Usually |
|  | Always |
|  | I don’t know |
|  |  |
| **5.** | **How often was the patient's pain well controlled?** |
|  | Never |
|  | Sometimes |
|  | Usually |
|  | Always |
|  | I don’t know |
|  |  |
| **6.** | **How often did the healthcare practitioners do everything they could to help with the patient's discomfort, agitation or irritability?** |
|  | Never |
|  | Sometimes |
|  | Usually |
|  | Always |
|  | I don’t know |

| **12.** | **Did a healthcare practitioner (e.g. nurse, social worker, psychologist) offer to speak with the patient about their mental or emotional health?** |
| --- | --- |
|  | No, but they did not need support |
|  | No and they needed support |
|  | Yes, but they did not need support |
|  | Yes, but they needed more support |
|  | Yes and they got all the support they needed |
|  | I don’t know |
|  |  |
| **13.** | **How often did the patient experience care**  **that was unsafe?** |
|  | Never |
|  | Sometimes |
|  | Usually |
|  | Always |
|  | I don’t know |
|  |  |
| **14.** | **How often was the patient treated unfairly because of their age, ethnicity, gender, cultural beliefs, religious beliefs, or other**  **personal characteristics?** |
|  | Never |
|  | Sometimes |
|  | Usually |
|  | Always |
|  | I don’t know |
|  |  |

| ***Overall Care*** |
| --- |
|  |

| **15.** | **Please provide an overall rating of the care**  **that the patient has received for this injury.** |
| --- | --- |
|  | 0 - Worst Injury Care Possible |
|  | 1 |
|  | 2 |
|  | 3 |
|  | 4 |
|  | 5 |
|  | 6 |
|  | 7 |
|  | 8 |
|  | 9 |
|  | 10 - Best Injury Care Possible |
|  |  |

| **16.** | **Since being injured, which of the following options best describes the patient’s current overall physical health?** |
| --- | --- |
|  | Excellent |
|  | Very good |
|  | Good |
|  | Fair |
|  | Poor |
|  |  |
| **17.** | **Since being injured, which of the following**  **options best describes the patient’s current**  **overall mental or emotional health?** |
|  | Excellent |
|  | Very good |
|  | Good |
|  | Fair |
|  | Poor |

| **18.** | **Please provide comments on how we can**  **improve injury care for patients.** |
| --- | --- |
|  | |
|  | |
|  | |
|  | |
|  | |
|  | |
|  | |
|  | |
|  | |
|  | |
|  | |

| **Demographic questions on next page** |
| --- |

| ***Questions about the patient*** | | | | | | | | | | | |
| --- | --- | --- | --- | --- | --- | --- | --- | --- | --- | --- | --- |
|  | | | | | | | | | | | |
| **19.** | **The patient would identify as...** | | | | | | | | | | |
|  | Male | | | | |  | | Female | | | |
|  |  | | | | | | | | | | |
| **20.** | **What is the patient’s age? (e.g., 40 years old)** | | | | | | | | | | |
|  | | | | | | | | | | | |
|  | | | | | | | | | | | |
| **21.** | **How was the patient injured?** | | | | | | | | | | |
|  | Car crash | | | | | | | | | | |
|  | ATV/off-roading vehicle crash | | | | | | | | | | |
|  | Pedestrian/bicycle hit by a motor vehicle | | | | | | | | | | |
|  | Bicycle crash | | | | | | | | | | |
|  | Fall | | | | | | | | | | |
|  | Assault | | | | | | | | | | |
|  | Burn | | | | | | | | | | |
|  | Self-harm | | | | | | | | | | |
|  | Other (please specify): | | | | | | | |  | | |
|  | | | | | | | | | | | |
| **22.** | | **What is the highest level of education that the patient has completed?** | | | | | | | | | |
|  | | 8th grade or less | | | | | | | | | |
|  | | Some high school, but did not graduate | | | | | | | | | |
|  | | High school or high school equivalency | | | | | | | | | |
|  | | Some college/university, did not graduate | | | | | | | | | |
|  | | College, CGEP, or other non-university certificate or diploma | | | | | | | | | |
|  | | University degree | | | | | | | | | |
|  | | Post-graduate degree or professional designation | | | | | | | | | |
|  | |  | | | | | | | | | |
| **23.** | **What language does the patient mainly speak at home?** | | | | | | | | | | |
|  | English | | | |  | | French | | | | |
|  | Other (please specify): | | | | | | | |  | | |
|  | | | | | | | | | | | |
| **24.** | **Does the patient consider them self to be…** | | | | | | | | | | |
|  | White | |  | Black | | | | | |  | Chinese |
|  | First Nations, Metis, Inuk, Aboriginal, or Indigenous | | | | | | | | | | |
|  | South Asian  (East Indian, Pakistani, Sri Lankan, etc.) | | | | | | | | | | |
|  | Other (please specify): | | | | | | | |  | | |

| ***Questions about you*** | | | | | | | | | | | | | |
| --- | --- | --- | --- | --- | --- | --- | --- | --- | --- | --- | --- | --- | --- |
|  | | | | | | | | | | | | | |
| **25.** | **I am...** | | | | | | | | | | | | |
|  | Male | | | | |  | Female | | | | | | |
|  |  | | | | | | | | | | | | |
| **26.** | **What is your age? (e.g., 40 years old)** | | | | | | | | | | | | |
|  | | | | | | | | | | | | | |
|  | | | | | | | | | | | | | |
| **27.** | **What language do you mainly speak at home?** | | | | | | | | | | | | |
|  | English | | | |  | French | | | | | | | |
|  | Other (please specify): | | | | | | |  | | | | | |
|  | | | | | | | | | | | | | |
| **28.** | | **On average, how often did you see the patient before his/her injury?** | | | | | | | | | | | |
|  | | More than once a week | | | | | | | | | | | |
|  | | Once a week | | | | | | | | | | | |
|  | | Monthly | | | | | | | | | | | |
|  | | Yearly | | | | | | | | | | | |
|  | | Less than once a year | | | | | | | | | | | |
|  | |  | | | | | | | | | | | |
| **29.** | | **What is the highest level of education that you have completed?** | | | | | | | | | | | |
|  | | 8th grade or less | | | | | | | | | | | |
|  | | Some high school, but did not graduate | | | | | | | | | | | |
|  | | High school or high school equivalency | | | | | | | | | | | |
|  | | Some college/university, did not graduate | | | | | | | | | | | |
|  | | College, CGEP, or other non-university certificate or diploma | | | | | | | | | | | |
|  | | University degree | | | | | | | | | | | |
|  | | Post-graduate degree or professional designation | | | | | | | | | | | |
| **30.** | | **Do you consider yourself to be…** | | | | | | | | | | | |
|  | | White |  | Black | | | | | |  | Chinese | | |
|  | | First Nations, Metis, Inuk, Aboriginal, or Indigenous | | | | | | | | | | | |
|  | | South Asian  (East Indian, Pakistani, Sri Lankan, etc.) | | | | | | | | | | | |
|  | | Other (please specify): | | | | | | |  | | | | |
|  | |  | | | | | | | | | | | |
| **31.** | | **I am the patient's…** | | | | | | | | | | | |
|  | | Wife |  | Husband | | | | | | | |  | Partner |
|  | | Mother |  | Grandmother | | | | | | | |  | |
|  | | Father |  | Grandfather | | | | | | | |  | |
|  | | Sister |  | Brother | | | | | | | |  | |
|  | | Son |  | Daughter | | | | | | | |  | |
|  | | Other (please specify): | | | | | | |  | | | | |
