# Supplementary material for: Short form version of the Quality of Trauma Care Patient-Reported Experience Measure (SF QTAC-PREM)
Source: BMC Res Notes. 2017 Dec 6;10:693. doi: 10.1186/s13104-017-3031-9 (PMC5718023; doi:10.1186/s13104-017-3031-9)
Supplement: Supplementary file 3 — Additional file 3. Quality of Trauma Care Patient-Reported Experience Measure (QTAC-PREM)—Short Form. Part 2: Post-Acute Care, Patient Survey. [file 13104_2017_3031_MOESM3_ESM.doc]

**Quality of Trauma Care Patient-Reported Experience Measure (QTAC-PREM) – Short Form**

**Part 2: Post-Acute Care, Patient Survey**

| ***Read*** |
| --- |
| - This survey contains questions about your discharge from hospital and about the follow-up appointments, visits, information, and care you have received since leaving the hospital. - I will ask you questions and read the response options to you. Please tell me the response option that best reflects your experience. - This survey will take about ten minutes to complete. - There are 15 questions. - When answering questions about discharge think about the last hospital that you were discharged from. |

| **1.** | **Did you receive written discharge instructions that provided you with enough information to help you care for your injuries?** |
| --- | --- |
|  | I did not receive written discharge instructions |
|  | I received written discharge instructions but I wanted more information |
|  | I received written discharge instructions and I got all the information I wanted |
|  |  |
| **2.** | **After being discharged from the hospital,**  **did you have enough pain medication to control your pain well?** |
| SHAPE \* MERGEFORMAT | Yes |
| SHAPE \* MERGEFORMAT | No |
| SHAPE \* MERGEFORMAT | Not applicable |
|  |  |
| **3.** | **Since being discharged from the hospital, have you attended an appointment or had a visit related to your injuries with…** |
|  |  |
| **A.** | **Family doctors, general practitioners, or doctors at walk-in clinics?** |
| SHAPE \* MERGEFORMAT | Yes |
| SHAPE \* MERGEFORMAT | No |
| **B.** | **Any other doctors including surgeons or specialists?** |
| SHAPE \* MERGEFORMAT | Yes |
| SHAPE \* MERGEFORMAT | No |
| **C.** | **Physiotherapists, rehabilitation therapists, or occupational therapist?** |
| SHAPE \* MERGEFORMAT | Yes |
| SHAPE \* MERGEFORMAT | No |
| **D.** | **Home care nurses or community nurses?** |
| SHAPE \* MERGEFORMAT | Yes |
| SHAPE \* MERGEFORMAT | No |
|  |  |
| **4.** | **Are you scheduled for an appointment or visit related to your injuries with…** |
|  |  |
| **A.** | **Family doctors, general practitioners, or doctors at walk-in clinics?** |
| SHAPE \* MERGEFORMAT | Yes |
| SHAPE \* MERGEFORMAT | No |
| **B.** | **Any other doctors including surgeons or specialists?** |
| SHAPE \* MERGEFORMAT | Yes |
| SHAPE \* MERGEFORMAT | No |
| **C.** | **Physiotherapists, rehabilitation therapists, or occupational therapist?** |
| SHAPE \* MERGEFORMAT | Yes |
| SHAPE \* MERGEFORMAT | No |
| **D.** | **Home care nurses or community nurses?** |
| SHAPE \* MERGEFORMAT | Yes |
| SHAPE \* MERGEFORMAT | No |

| **5.** | **Have you had difficulty scheduling appointments or visits that are convenient for you?** | | |
| --- | --- | --- | --- |
| SHAPE \* MERGEFORMAT | No | | Go to question 7 |
| SHAPE \* MERGEFORMAT | Yes, slight difficulty | | Go to question 6 |
|  | Yes, moderate difficulty | | Go to question 6 |
| SHAPE \* MERGEFORMAT | Yes, severe difficulty | | Go to question 6 |
|  |  | |  |
| **6.** | **With which of the following practitioners have you had difficulty scheduling follow-up appointments or visits?** | | |
|  |  | | |
| **A.** | **Family doctors, general practitioners, or doctors at walk-in clinics?** | | |
| SHAPE \* MERGEFORMAT | Yes | | |
| SHAPE \* MERGEFORMAT | No | | |
| **B.** | **Any other doctors including surgeons or specialists?** | | |
| SHAPE \* MERGEFORMAT | Yes | | |
| SHAPE \* MERGEFORMAT | No | | |
| **C.** | **Physiotherapists, rehabilitation therapists, or occupational therapist?** | | |
| SHAPE \* MERGEFORMAT | Yes | | |
| SHAPE \* MERGEFORMAT | No | | |
| **D.** | **Home care nurses or community nurses?** | | |
| SHAPE \* MERGEFORMAT | Yes | | |
| SHAPE \* MERGEFORMAT | No | | |
|  |  | | |
| ***Question Guide #1*** | | | |
| If "No" answered for all of 3 and 4, skip to question 11. | | | |
| ***Read*** | | | |
| I’m now going to ask you about your follow-up appointments and visits. These questions apply to all of the healthcare professionals that you have seen since being discharged from the hospital. | | | |
|  | | | |
| **7.** | **During your follow-up appointments or visits,**  **did your healthcare practitioners explain the steps involved in your recovery from injury for example, activities you should or should not do, necessary medications, tests and treatments, or other follow-up appointments?** | | |
| SHAPE \* MERGEFORMAT | No | | |
| SHAPE \* MERGEFORMAT | Yes, but I wanted more information | | |
| SHAPE \* MERGEFORMAT | Yes and I got all the information I wanted | | |
|  |  | | |
| **8.** | | **During your follow-up appointments or visits, did your healthcare practitioners explain approximately how long it would take you to recover?** | |
|  | | No | |
| SHAPE \* MERGEFORMAT | | Yes, but I wanted more information | |
| SHAPE \* MERGEFORMAT | | Yes and I got all the information I wanted | |
| **9.** | | **During your follow-up appointments or visits, how often did your healthcare practitioners explain things in a way you could understand?** | |
| SHAPE \* MERGEFORMAT | | Never | |
| SHAPE \* MERGEFORMAT | | Sometimes | |
| SHAPE \* MERGEFORMAT | | Usually | |
| SHAPE \* MERGEFORMAT | | Always | |
|  | |  | |
| **10.** | | **Did your family physician or general practitioner receive information from the hospital about your injuries, your hospital stay, or the care you would need to continue your recovery?** | |
| SHAPE \* MERGEFORMAT | | No | |
| SHAPE \* MERGEFORMAT | | Yes, but they wanted more information | |
| SHAPE \* MERGEFORMAT | | Yes and they got all the information they wanted | |
|  | | I don’t know | |
|  | | I haven’t seen a family physician or general practitioner since being discharged | |
|  | |  | |
| **11.** | | **Since being discharged from the hospital, how often have you experienced healthcare that was unsafe?** | |
| SHAPE \* MERGEFORMAT | | Never | |
| SHAPE \* MERGEFORMAT | | Sometimes | |
| SHAPE \* MERGEFORMAT | | Usually | |
| SHAPE \* MERGEFORMAT | | Always | |
|  | |  | |
| **12.** | | **On a scale of zero to ten, how well have you been guided through the recovery process by your healthcare practitioners since being discharged from the hospital, zero being poor guidance, ten being excellent guidance.** | |
| SHAPE \* MERGEFORMAT | | 0 - Poor Guidance | |
|  | | 1 | |
| SHAPE \* MERGEFORMAT | | 2 | |
| SHAPE \* MERGEFORMAT | | 3 | |
|  | | 4 | |
| SHAPE \* MERGEFORMAT | | 5 | |
| SHAPE \* MERGEFORMAT | | 6 | |
| SHAPE \* MERGEFORMAT | | 7 | |
| SHAPE \* MERGEFORMAT | | 8 | |
|  | | 9 | |
| SHAPE \* MERGEFORMAT | | 10 - Excellent Guidance | |

| **13.** | **On a scale of zero to ten, please provide an overall rating of the follow-up care, appointments, visits, and information you have received so far since being discharged from the hospital, zero being the worst injury care possible, ten being the best injury care possible.** | |
| --- | --- | --- |
| SHAPE \* MERGEFORMAT | 0 - Worst Injury Care Possible | |
| SHAPE \* MERGEFORMAT | 1 | |
| SHAPE \* MERGEFORMAT | 2 | |
| SHAPE \* MERGEFORMAT | 3 | |
| SHAPE \* MERGEFORMAT | 4 | |
| SHAPE \* MERGEFORMAT | 5 | |
| SHAPE \* MERGEFORMAT | 6 | |
| SHAPE \* MERGEFORMAT | 7 | |
| SHAPE \* MERGEFORMAT | 8 | |
| SHAPE \* MERGEFORMAT | 9 | |
|  | 10 - Best Injury Care Possible | |
|  |  | |
| **14.** | | **Which of the following options best describes your current overall physical health?** |
|  | | Excellent |
| SHAPE \* MERGEFORMAT | | Very good |
| SHAPE \* MERGEFORMAT | | Good |
| SHAPE \* MERGEFORMAT | | Fair |
| SHAPE \* MERGEFORMAT | | Poor |
|  | |  |
| **15.** | **Which of the following options best describes your current overall mental or emotional health?** | |
| SHAPE \* MERGEFORMAT | Excellent | |
| SHAPE \* MERGEFORMAT | Very good | |
| SHAPE \* MERGEFORMAT | Good | |
| SHAPE \* MERGEFORMAT | Fair | |
| SHAPE \* MERGEFORMAT | Poor | |
| ***Read*** | | |
| The last question is open ended. I will read the question to you and write down what you say**.** | | |
|  | | |
| **16.** | | **Please provide comments on how we can improve injury care for patients after they are discharged from hospital?** |
|  | | |
|  | | |
|  | | |

| ***Read*** |
| --- |
| That’s the end of the survey. Thank you so much for taking the time to speak with me. |
