# Supplementary material for: Short form version of the Quality of Trauma Care Patient-Reported Experience Measure (SF QTAC-PREM)
Source: BMC Res Notes. 2017 Dec 6;10:693. doi: 10.1186/s13104-017-3031-9 (PMC5718023; doi:10.1186/s13104-017-3031-9)
Supplement: Supplementary file 4 — Additional file 4. Quality of Trauma Care Patient-Reported Experience Measure (QTAC-PREM)—Short Form. Part 2: Post-Acute Care, Family Member/Proxy Survey. [file 13104_2017_3031_MOESM4_ESM.docx]

**Quality of Trauma Care Patient-Reported Experience Measure (QTAC-PREM) – Short Form**

**Part 2: Post-Acute Care, Family Member/Proxy Survey**

| ***Read*** |
| --- |
| - This survey contains questions about the patient’s discharge from hospital and about the follow-up appointments, visits, information, and care the patient has received since leaving the hospital. - When completing this survey remember that you are completing it for the patient. - Think about how the patient would answer the questions. - I will ask you questions and read the response options to you. Please tell me the response option that best reflects how the patient would answer. - This survey will take about ten minutes to complete. - There are 15 questions. - When answering questions about discharge think about the last hospital that the patient was discharged from. |

| **1.** | **Did the patient receive written discharge instructions that provided them with enough information to help them care for their injuries?** |
| --- | --- |
|  | They did not receive written discharge instructions |
|  | They received written discharge instructions but wanted more information |
|  | They received written discharge instructions and got all the information they wanted |
|  | I don’t know |
|  |  |
| **2.** | **After being discharged from the hospital,**  **did the patient have enough pain medication to control their pain well?** |
|  | Yes |
|  | No |
|  | Not applicable |
|  | I don’t know |
|  |  |
| **3.** | **Since being discharged from the hospital, has the patient attended an appointment or had a visit related to their injuries with…** |
|  |  |
| **A.** | **Family doctors, general practitioners, or doctors at walk-in clinics?** |
|  | Yes |
|  | No |
|  | I don’t know |
| **B.** | **Any other doctors including surgeons or specialists?** |
|  | Yes |
|  | No |
|  | I don’t know |
| **C.** | **Physiotherapists, rehabilitation therapists, or occupational therapist?** |
|  | Yes |
|  | No |
|  | I don’t know |
| **D.** | **Home care nurses or community nurses?** |
|  | Yes |
|  | No |
|  | I don’t know |
|  |  |

| **4.** | **Is the patient scheduled for an appointment or visit related to their injuries with…** |
| --- | --- |
|  |  |
| **A.** | **Family doctors, general practitioners, or doctors at walk-in clinics?** |
|  | Yes |
|  | No |
|  | I don’t know |
| **B.** | **Any other doctors including surgeons or specialists?** |
|  | Yes |
|  | No |
|  | I don’t know |
| **C.** | **Physiotherapists, rehabilitation therapists, or occupational therapist?** |
|  | Yes |
|  | No |
|  | I don’t know |
| **D.** | **Home care nurses or community nurses?** |
|  | Yes |
|  | No |
|  | I don’t know |

| **5.** | **Has the patient had difficulty scheduling appointments or visits that are convenient for them?** | |
| --- | --- | --- |
|  | No | Go to question 7 |
|  | Yes, slight difficulty | Go to question 6 |
|  | Yes, moderate difficulty | Go to question 6 |
|  | Yes, severe difficulty | Go to question 6 |
|  | I don’t know |  |
|  |  |  |
| **6.** | **With which of the following practitioners has the patient had difficulty scheduling follow-up appointments or visits?** | |
|  |  | |
| **A.** | **Family doctors, general practitioners, or doctors at walk-in clinics?** | |
|  | Yes | |
|  | No | |
|  | I don’t know | |
| **B.** | **Any other doctors including surgeons or specialists?** | |
|  | Yes | |
|  | No | |
|  | I don’t know | |
| **C.** | **Physiotherapists, rehabilitation therapists, or occupational therapist?** | |
|  | Yes | |
|  | No | |
|  | I don’t know | |
| **D.** | **Home care nurses or community nurses?** | |
|  | Yes | |
|  | No | |
|  | I don’t know | |

| ***Question Guide #1*** | | | | |  |
| --- | --- | --- | --- | --- | --- |
| If "No" answered for all of 3 and 4, skip to question 10. | | | | |  |
| ***Read*** | | | | |  |
| I’m now going to ask you about the patient’s follow-up appointments and visits. These questions apply to all of the healthcare professionals that the patient has seen since being discharged from the hospital. | | | | |  |
|  | | | | |  |
| **7.** | **During the patients’ follow-up appointments or visits, did their healthcare practitioners explain the steps involved in their recovery from injury for example, activities they should or should not do, necessary medications, tests and treatments, or other follow-up appointments?** | | | |  |
|  | No | | | |  |
|  | Yes, but the patient wanted more information | | | |  |
|  | Yes and the patient got all the information they wanted | | | |  |
|  | I don’t know | | | |  |
|  |  | | | |  |
| **8.** | | **During the patient’s follow-up appointments or visits, did their healthcare practitioners explain approximately how long it would take them to recover?** | | |  |
|  | | No | | |  |
|  | | Yes, but the patient wanted more information | | |  |
|  | | Yes and the patient got all the information they wanted | | |  |
|  | | I don’t know | | |  |
|  | |  | | |  |
| **9.** | | **During the patient’s follow-up appointments or visits, how often did their healthcare practitioners explain things in a way they could understand?** | | |  |
|  | | Never | | |  |
|  | | Sometimes | | |  |
|  | | Usually | | |  |
|  | | Always | | |  |
|  | | I don’t know | | |  |
|  | |  | | |  |
| **10.** | | **Did the patient’s family physician or general practitioner receive information from the hospital about their injuries, their hospital stay, or the care they would need to continue their recovery?** | | |  |
|  | | No | | |  |
|  | | Yes, but they wanted more information | | |  |
|  | | Yes and they got all the information they wanted | | |  |
|  | | I don’t know | | |  |
|  | | The patient hasn’t seen a family physician or general practitioner since being discharged | | |  |
| **10.** | | **Since being discharged from the hospital, how often has the patient experienced healthcare that was unsafe?** | | |  |
|  | | Never | | |  |
|  | | Sometimes | | |  |
|  | | Usually | | |  |
|  | | Always | | |  |
|  | | I don’t know | | |  |
|  | |  | | |  |
| **11.** | | **On a scale of zero to ten, how well has the patient been guided through the recovery process by their healthcare practitioners since being discharged from the hospital, zero being poor guidance, ten being excellent guidance.** | | |  |
|  | | 0 - Poor Guidance | | |  |
|  | | 1 | | |  |
|  | | 2 | | |  |
|  | | 3 | | |  |
|  | | 4 | | |  |
|  | | 5 | | |  |
|  | | 6 | | |  |
|  | | 7 | | |  |
|  | | 8 | | |  |
|  | | 9 | | |  |
|  | | 10 - Excellent Guidance | | |  |
|  | |  | | |  |
| **12.** | | **On a scale of zero to ten, please provide an overall rating of the follow-up care, appointments, visits, and information the patient has received so far since being discharged from the hospital, zero being the worst injury care possible, ten being the best injury care possible.** | | |  |
|  | | 0 - Worst Injury Care Possible | | |  |
|  | | 1 | | |  |
|  | | 2 | | |  |
|  | | 3 | | |  |
|  | | 4 | | |  |
|  | | 5 | | |  |
|  | | 6 | | |  |
|  | | 7 | | |  |
|  | | 8 | | |  |
|  | | 9 | | |  |
|  | | 10 - Best Injury Care Possible | | |  |
|  | |  | | |  |
| **13.** | | | | **Which of the following options best describes the patient’s current overall physical health?** | |
|  | | | | Excellent | |
|  | | | | Very good | |
|  | | | | Good | |
|  | | | | Fair | |
|  | | | | Poor | |
|  | | | |  | |
| **14.** | | **Which of the following options best describes the patient’s current overall mental or emotional health?** | | | |
|  | | Excellent | | | |
|  | | Very good | | | |
|  | | Good | | | |
|  | | Fair | | | |
|  | | Poor | | | |
| ***Read*** | | | | | |
| The last question is open ended. I will read the question to you and write down what you say**.** | | | | | |
|  | | | | | |
| **15.** | | | **Please provide comments on how we can improve injury care for patients after they are discharged from hospital?** | | |
|  | | | | | |
|  | | | | | |
|  | | | | | |
|  | | | | | |
|  | | | | | |
|  | | | | | |
|  | | | | | |
|  | | | | | |
|  | | | | | |
|  | | | | | |

| ***Read*** |
| --- |
| That’s the end of the survey. Thank you so much for taking the time to speak with me. |
