# Supplementary material for: Short form version of the Quality of Trauma Care Patient-Reported Experience Measure (SF QTAC-PREM)
Source: BMC Res Notes. 2017 Dec 6;10:693. doi: 10.1186/s13104-017-3031-9 (PMC5718023; doi:10.1186/s13104-017-3031-9)
Supplement: Supplementary file 5 — Additional file 5. Mapping the derivation of the short form acute care QTAC-PREM from the original long form items. [file 13104_2017_3031_MOESM5_ESM.docx]

| Additional File 1. Mapping the derivation of the short form acute care QTAC-PREM from the original long form items | | |
| --- | --- | --- |
| **Long Form Item**   - Response options | **Short Form Item**   - Response options | **Reason and Supporting Statistics**  Item maintained; item revised; responses revised; deleted |
| **1. What is your sex?**   - Female - Male | **19. What is your gender?**   - Female - Male | **Item revised.**  Allows participants to identify with their self-identified gender, not constrained by biological sex. |
| **2. What is your age?**   - *Free text* | **20. What is your age?**   - *Free text* | **Item maintained.** |
| **3. Where do you live (city/town, province)?**   - *Free text* |  | **Deleted**.  Non-essential demographic information. |
| **4. How were you injured?**   - Car crash - Pedestrian-vehicle - Bicycle - Fall - Assault - Burn - Attempted suicide - Other (please specify): *free text* | **21. How were you injured?**   - Car crash - ATV/off-roading vehicle crash - Bicycle - Fall - Assault - Burn - Self-harm - Other (please specify): *free text* | **Responses revised.**  A large portion of participants in the validation study described injuries in the free text “other” option which were not captured by the check box responses. |
| **5. What is the highest level of education you have completed?**   - 8th grade or less - Some high school, but did not graduate - High school graduate - Some college/university, did not graduate - College diploma/certificate - 4 year university degree - More than 4 year university degree | **23. What is the highest level of education that you have completed?**   - 8th grade or less - Some high school, but did not graduate - High school or high school equivalency - Some college/university, did not graduate - College, CGEP, or other non-university certificate or diploma - University degree - Post-graduate degree or professional designation | **Responses revised.**  Options revised to match Canadian Institute of Health Information patient experience survey. |
| **6. What is your ethnicity?**   - Caucasian - Black or African Canadian - Asian - Aboriginal or First Nations - East Indian - Latin American - Other (please print): *free text* | **24. Do you consider yourself to be…**   - White - Chinese - First Nations, Metis, Inuk, Aboriginal, or Indigenous - South Asian (East Indian, Pakistani, Sri Lankan, etc.) - Black - Other (please specify): *free text* | **Item revised. Responses revised.**  Options revised to match Canadian Institute of Health Information patient experience survey. Also informed by most common responses in the validation study. |
| **7. What language do you mainly speak at home?**   - English - French - Vietnamese - Spanish - Chines   (Mandarin/Cantonese)   - Other (please print): *free text* | **22. What language do you mainly speak at home?**   - English - French - Other (please print): *free text* | **Responses revised.**  Options revised to the two official languages of Canada. |
| **8. Since being injured, which of the following options best describes your overall health?**   - Excellent - Very good - Good - Fair - Poor | **16. Since being injured, which of the following options best describes your current overall physical health?**   - Excellent - Very good - Good - Fair - Poor   **17. Since being injured, which of the following options best describes your current overall mental or emotional health?**   - Excellent - Very good - Good - Fair - Poor | **Item revised.**  Item split into two items. Revised to match the Canadian Institute of Health Information patient experience survey. Allows for assessment of physical and mental health. |
| **9**. **How often did your healthcare providers (e.g. doctors, nurses, therapists, etc.) explain things in a way you could understand?**   - Never - Sometimes - Usually - Always |  | **Deleted.**  Item covered by the HCAPS and CIHI patient experience surveys. |
| **10. Did your healthcare providers clearly**  **explain all your injuries to you in a way**  **you could understand?**   - Yes - No - Not able to answer | **1. Did your healthcare practitioners clearly explain all your injuries to you in a way you could understand?**   - No - Yes, but I wanted more information - Yes, and I got all the information I wanted | **Responses revised.**  The three-point scale may provide greater spread in the response distribution, decrease collinearity with similar questions, and would allow mapping of responses to the frequency response options used for the rest of the survey (low quality [never sometimes]; medium quality [usually]; high quality [always]). We added a component to the response options to assess the adequacy of information. Many patients reported receiving information but in the free-text item reported that the information was insufficient and they wanted more information. |
| **11. Did your healthcare providers discuss how the injuries might affect you after you leave the hospital?**   - Yes - No - Not able to answer |  | **Deleted.**  Content covered by long form items 12 and 13, which asked about more tangible and specific events. |
| **12. Did your healthcare providers give**  **instructions on how you should care for**  **your injuries after you leave the hospital?**   - Yes - No - Not able to answer | **2. Did your healthcare practitioners give instructions on how you should care for your injuries?**   - No - Yes, but I wanted more information - Yes, and I got all the information I wanted | **Responses revised.**  Slightly shortened: no longer specified after leaving hospital. Item made more general. Response options changed. The three-point scale may provide greater spread in the response distribution, decrease collinearity with similar questions, and would allow mapping of responses to the frequency response options used for the rest of the survey (low quality [never sometimes]; medium quality [usually]; high quality [always]). We added a component to the response options to assess the adequacy of information. Many patients reported receiving information but in the free-text item reported that the information was insufficient and they wanted more information. |
| **13. Did your healthcare providers discuss how long it might take you to recover from your injuries?**   - Yes - No - Not able to answer | **3. Did your healthcare practitioners discuss how long it might take you to recover from your injuries?**   - No - Yes, but I wanted more information - Yes, and I got all the information I wanted | **Responses revised.**  Response options changed. The three-point scale may provide greater spread in the response distribution, decrease collinearity with similar questions, and would allow mapping of responses to the frequency response options used for the rest of the survey (low quality [never sometimes]; medium quality [usually]; high quality [always]). We added a component to the response options to assess the adequacy of information. Many patients reported receiving information but in the free-text item reported that the information was insufficient and they wanted more information. |
| **14. How often was the information you**  **received from your various healthcare providers consistent?**   - Never - Sometimes - Usually - Always | **4. How often was the information you received from your various healthcare practitioners consistent?**   - Never - Sometimes - Usually - Always | **Item maintained.** |
| **15.** **During your transfers did the hospital staff or healthcare providers clearly explain where you were being transferred to?**   - Yes - No - I don’t know |  | **Deleted.**  Low correlation with the overall rating of quality (0.16 correlation). Limited response variability. Not able to factor analyse. |
| **16.** **During your transfers, how often were you kept comfortable?**   - Never - Sometimes - Usually - Always |  | **Deleted.**  Low correlation with global rating (0.36 correlation). Content overlap with long form item 18 (pain, 0.27 correlation) and long form item 22 (moved carefully, 0.42 correlation). Item 22 had a higher correlation with the global rating (0.36 vs. 0.44 correlation). |
| **17. When you arrived to a new hospital unit did a healthcare provider explain where important landmarks were in the unit? (e.g. call button, bathroom,**  **nurse’s station, water/ice machine)**   - Yes - No - I don’t know |  | **Deleted.**  Low correlation with the global rating (0.31 correlation). |
| **18. How often was your pain well controlled?**   - Never - Sometimes - Usually - Always - Not Applicable- did not have pain | **5. How often was your pain well controlled?**   - Never - Sometimes - Usually - Always | **Responses revised.**  Not application option deleted. Assumption is that all hospitalised injury patients will experience some pain. Elimination of the not application option simplifies analysis. |
| **19. How often did the healthcare providers do everything they could to help you with your pain?**   - Never - Sometimes - Usually - Always - Not Applicable- did not have pain |  | **Deleted.**  Collinearity between items 18 and 19 (0.77 correlation). Item 18 performed better: higher test-retest reliability coefficient. |
| **20. How often did the healthcare providers do everything they could to help you with your difficulty breathing?**   - Never - Sometimes - Usually - Always - Not Applicable- no difficulty breathing |  | **Deleted.**  High “not applicable” response rate (36.75%). Collinear with item 21 (0.68 correlation), item 22 (0.82 correlation), and item 23 (0.66 correlation). Could not factor analyse. |
| **21. How often did the healthcare providers do everything they could to help you with your agitation or irritability?**   - Never - Sometimes - Usually - Always - Not Applicable- no agitation or irritability | **6. How often did the healthcare practitioners**  **do everything they could to help you with your discomfort, agitation or irritability?**   - Never - Sometimes - Usually - Always | **Item revised.**  Added the adverb “discomfort” to better describe the construct. Not application option deleted. Assumption is that all hospitalised injury patients will experience some discomfort. Elimination of the not application option simplifies analysis. |
| **22. When the healthcare providers rolled you, turned you over in bed, or helped you get out of bed and move around, how often did they do it carefully?**   - Never - Sometimes - Usually - Always - Not Applicable- did not need help moving | **7. When the healthcare practitioners helped you to move around (i.e., change position in bed, walking etc.) how often did they do it carefully?**   - Never - Sometimes - Usually - Always | **Item revised.**  Item slightly reworded to apply more broadly than just changing position or getting in or out of bed. |
| **23. How often did your nurses or other**  **hospital staff help you to maintain your**  **personal hygiene?**   - Never - Sometimes - Usually - Always - Not Applicable- did not need/want help | **8. How often did the** **hospital staff offer to help you maintain your personal hygiene?**   - Never - Sometimes - Usually - Always | **Item revised.**  List of staff members amalgamated to make shorter and simpler. |
| **24. When meeting a new healthcare provider for the first time how often did they introduce themselves and clearly explain their role in your care?**   - Never - Sometimes - Usually - Always | **9. When meeting a new healthcare practitioners for the first time how often did they introduce themselves and clearly explain their role in your care?**   - Never - Sometimes - Usually - Always | **Item maintained.** |
| **25. When you expressed concerns or**  **frustrations about your care how often did your healthcare providers take action to deal with them?**   - Never - Sometimes - Usually - Always - Not Applicable- had no concerns | **10.** **When you had questions, concerns, or frustrations about your care how often did your healthcare practitioners take action?**   - Never - Sometimes - Usually - Always | **Item revised. Responses revised.**  Slightly revised by adding the word “question” in order to eliminate the “not applicable” option. Assumption is that all patients will have had a question about their care. |
| **26. Did a healthcare staff member**  **(e.g. psychologist, social worker, nurse) offer to speak with you about your emotional needs?**   - Yes - No | **12. Did a healthcare practitioner (e.g. nurse, social worker, psychologist) offer to speak with you about your mental or emotional health?** | **Item revised. Responses revised.**  Slightly revised to probe the construct of ancillary support and mental health as opposed to emotional needs. Patients during the validation study commented that they answered “no” to the item because they felt the didn’t have any emotional needs. This revised response scale will identify unmet support needs and provide increased response distribution. |
| **27. How often was your dignity considered by the healthcare providers?**   - Never - Sometimes - Usually - Always | **11.** **How often did your healthcare practitioners treat you with dignity?**   - Never - Sometimes - Usually - Always | **Item revised.**  Revision addresses a higher standard of quality. “Considering” a patient’s dignity may not be a high enough standard- patients ought to be “treated” with dignity. |
| **28. How often did you experience care that was unsafe?**   - Never - Sometimes - Usually - Always | **13.** **How often did you experience care that was unsafe?**   - Never - Sometimes - Usually - Always | **Item maintained.** |
| **29. How often were your cultural, religious, or spiritual preferences respected by the healthcare staff and religious or spiritual staff?**   - Never - Sometimes - Usually - Always - Not Applicable |  | **Deleted.**  High “not applicable” response rate. Low correlation with overall rating of quality (0.25 correlation). Could not factor analyse. |
| **30. How often were you treated unfairly**  **because of your age, ethnicity, gender, or personal characteristics?**   - Never - Sometimes - Usually - Always | **14. How often were you treated unfairly**  **because of your age, ethnicity, gender, cultural beliefs, religious beliefs, or personal characteristics?**   - Never - Sometimes - Usually - Always | **Item revised.**  Cultural and religious beliefs added in as these are also sources of possible discrimination. |
| **31. Please provide an overall rating of the care you have received for this injury.**   - 0 Worst injury care possible - 1 - 2 - 3 - 4 - 5 - 6 - 7 - 8 - 9 - 10 Best injury care possible | **15.** **Please provide an overall rating of the care**  **you have received for this injury.**   - 0 Worst injury care possible - 1 - 2 - 3 - 4 - 5 - 6 - 7 - 8 - 9 - 10 Best injury care possible | **Item maintained.** |
| **32. What was the best aspect of the care you received?**   - *Free text* |  | **Deleted.**  To reduce item count the measure will only include one free text item. Most patients duplicated comments between free text items. |
| **33. What was the worst aspect of the care you received?**  *Free text* |  | **Deleted.**  To reduce item count the measure will only include one free text item. Most patients duplicated comments between free text items. |
| **34. Describe any care you received that was unsafe.**  *Free text* |  | **Deleted.**  To reduce item count the measure will only include one free text item. Most patients duplicated comments between free text items. |
| **35. Provide comments on how we can**  **improve injury care for patients.**  *Free text* | **18. Provide comments on how we can**  **improve injury care for patients.**  *Free text* | **Item maintained.** |
