# Supplementary material for: Short form version of the Quality of Trauma Care Patient-Reported Experience Measure (SF QTAC-PREM)
Source: BMC Res Notes. 2017 Dec 6;10:693. doi: 10.1186/s13104-017-3031-9 (PMC5718023; doi:10.1186/s13104-017-3031-9)
Supplement: Supplementary file 6 — Additional file 6. Mapping the derivation of the short form post-acute care QTAC-PREM from the original long form items. [file 13104_2017_3031_MOESM6_ESM.docx]

| Additional File 2. Mapping the derivation of the short form post-acute care QTAC-PREM from the original long form items | | |
| --- | --- | --- |
| **Long Form Item**   - Response options | **Short Form Item**   - Response options | **Reason and Supporting Statistics**  Item maintained; item revised; responses revised; deleted |
| **1. Where did you go after being discharged from [*hospital name]*?**   - Home (go to text box 3) - A friend or family member’s home (go to text box 3) - Another hospital (go to text box 2) - An in-patient rehabilitation facility (go to question 6) - A long term care facility (go to question 6) |  | **Deleted**  We recommend this survey be used in patients that are being discharged to the community. Therefore, determining discharge disposition is not necessary. Community discharged patients composed the vast majority of respondents in our validation study. Based on formal and informal qualitative feedback we received when completing the post-acute survey via telephone it seems that patients going to long term care facilities face a different set of issues than community discharged patients. Patients discharged to another hospital or an in-patient rehabilitation facility for a short stay could be followed-up by partnering with those institutions to determine when patients are discharged back to the community. |
| **2. Before leaving the hospital, did your hospital healthcare providers give you written instructions on how to care for your injuries after being discharged?**   - Yes (go to question 3) - No (go to question 4) - Not Applicable (go to question 4) | **1. Did you receive written discharge instructions that provided you with enough information to help you care for your injuries?**   - I did not receive written discharge instructions - I received written discharge instructions but I wanted more information - I received written discharge instructions and I got all the information I wanted | **Item revised. Responses revised.**  This item was originally a screener item for item 3. We have combined the two items and modified the response options to form a single three-level ordinal variable. The screener item introduced challenges in data analysis as a large proportion of patients reportedly did not received discharge instructions. We also added a component to the response options to assess the adequacy of information. Many patients reported receiving information but in the free-text item reported that the information was insufficient and they wanted more information. |
| **3. Did the written instructions you received provide you with enough information to help you care for your injuries after being discharged?**   - Yes - No - Not Applicable | **1. Did you receive written discharge instructions that provided you with enough information to help you care for your injuries?**   - I did not receive written discharge instructions - I received written discharge instructions but I wanted more information - I received written discharge instructions and I got all the information I wanted | **Item revised. Responses revised.**  This item was originally a screener item for item 3. We have combined the two items and modified the response options to form a single three-level ordinal variable. The screener item introduced challenges in data analysis as a large proportion of patients reportedly did not received discharge instructions. We also added a component to the response options to assess the adequacy of information. Many patients reported receiving information but in the free-text item reported that the information was insufficient and they wanted more information. |
| **4. After being discharged from the hospital, did you have enough pain medication to control your pain well?**   - Yes - No - Not Applicable | **2. After being discharged from the hospital,**  **did you have enough pain medication to control your pain well?**   - Yes - No - Not Applicable | **Item maintained.** |
| **5. After being discharged from the hospital, did you get the all of the support services that you wanted, for example, home care, social**  **work, or counselling?**   - Yes - No - Not Applicable |  | **Deleted.**  High “not applicable” response rate (34%). |
| **6. Since being discharged from the hospital, have you attended an appointment to follow-up about your injuries with...**   1. A trauma doctor, surgeon, or specialist?  - Yes - No - Not Applicable  1. A family doctor or general practitioner?  - Yes - No - Not Applicable  1. A physio, rehabilitation or occupational therapist?  - Yes - No - Not Applicable | **3.** **Since being discharged from the hospital, have you attended an appointment or had a visit related to your injuries with…**  A. Family doctors, general practitioners, or doctors at walk-in clinics?   - Yes - No   B. Any other doctors including surgeons or specialists?   - Yes - No   C. Physiotherapists, rehabilitation therapists, or occupational therapist?   - Yes - No   D. Home care nurses or community nurses?   - Yes - No | **Item revised. Responses revised.**  During the telephone interviews participants commented that home care and community curses weren’t included on the list. They also mentioned other specialist physicians including neurosurgeons, neurologists, psychiatrists, nephrologist, etc. Therefore, responses were re-ordered and revised to reflect all the practitioners people may see post-discharge. We’ve also removed the not applicable option because it is not necessary. We also added “visit” into the wording as some participants commented that they didn’t perceive home visits as an appointment. |
| **7. Are you scheduled or planning to schedule an appointment to follow-up about your injuries with…**   1. A trauma doctor, surgeon, or specialist?  - Yes - No - Not Applicable  1. A family doctor or general practitioner?  - Yes - No - Not Applicable  1. A physio, rehabilitation or occupational therapist?  - Yes - No - Not Applicable | **4.** **Are you scheduled for an appointment or visit related to your injuries with…**  A. Family doctors, general practitioners, or doctors at walk-in clinics?   - Yes - No   B. Any other doctors including surgeons or specialists?   - Yes - No   C. Physiotherapists, rehabilitation therapists, or occupational therapist?   - Yes - No   D. Home care nurses or community nurses?   - Yes - No | **Item revised. Responses revised.**  During the telephone interviews participants commented that home care and community curses weren’t included on the list. They also mentioned other specialist physicians including neurosurgeons, neurologists, psychiatrists, nephrologist, etc. Therefore, responses were re-ordered and revised to reflect all the practitioners people may see post-discharge. We’ve removed the not applicable option because it is not necessary. We added “visit” into the wording as some participants commented that they didn’t perceive home visits as an appointment. We also removed the concept of “planning to schedule an appointment” in order to focus the item on organised appointments. |
| **8. Did you have difficulty getting follow-up appointments when you wanted them with…**   1. A trauma doctor, surgeon, or specialist?  - Yes - No - Not Applicable  1. A family doctor or general practitioner?  - Yes - No - Not Applicable  1. A physio, rehabilitation or occupational therapist?  - Yes - No - Not Applicable | **5. Have you had difficulty scheduling appointments or visits that are convenient for you?**   - No (go to question 7) - Yes, slight difficulty (go to question 6) - Yes, moderate difficulty (go to question 6) - Yes, severe difficulty (go to question 6)   **6. With which of the following providers have you had difficulty scheduling follow-up appointments or visits?**  A. Family doctors, general practitioners, or doctors at walk-in clinics?   - Yes - No   B. Any other doctors including surgeons or specialists?   - Yes - No   C. Physiotherapists, rehabilitation therapists, or occupational therapist?   - Yes - No   D. Home care nurses or community nurses?   - Yes - No | **Item revised. Responses revised.**  Item split into two items. Participants pursued follow-up with different combinations of practitioners. As a result, 57% responded “not applicable” to at least one of the items and this presented challenges in data analysis. We decided to simplify and create two items that would apply to all patients that answer them: one which examines challenges in pursuing following-up and, if participants answer yes, a second item that identifies the practitioner for which follow-up was difficult to schedule. Both items provide valuable information and this approach allows the elimination of the “not applicable” response option. We also changed the tense of the item because care was ongoing for many of the participants in the validation study. |
| **9. During your follow-up appointments, did your healthcare providers explain the next steps in your recovery from injury for example, activities you should or should not do, necessary medications, tests and treatments, or other follow-up appointments?**   - Yes - No - Not Applicable | **7. During your follow-up appointments or visits,**  **did your healthcare providers explain the steps involved in your recovery from injury for example, activities you should or should not do, necessary medications, tests and treatments, or other follow-up appointments?**   - No - Yes, but I wanted more information - Yes, and I got all the information I wanted | **Item revised. Responses revised.**  We added “visit” into the wording as some participants commented that they didn’t perceive home visits as an appointment.  We added a component to the response options to assess the adequacy of information. Many patients reported receiving information but in the free-text item reported that the information was insufficient and they wanted more information. |
| **10. During your follow-up appointments, did your healthcare providers explain approximately how long it would take you to**  **recover?**   - Yes - No - Not Applicable | **8.** **During your follow-up appointments or visits, did your healthcare providers explain approximately how long it would take you to recover?**   - No - Yes, but I wanted more information - Yes, and I got all the information I wanted | **Item revised. Responses revised.**  We added a component to the response options to assess the adequacy of information. Many patients reported receiving information but in the free-text item reported that the information was insufficient and they wanted more information.  We added “visit” into the wording as some participants commented that they didn’t perceive home visits as an appointment. |
| **11. During your follow-up appointments, did you get all of the information that you wanted from the healthcare providers?**   - Yes - No - Not Applicable |  | **Deleted.**  Adequacy and completeness of the information received was built into the response options of the information items. |
| **12. During your follow-up appointments, how often did your healthcare providers explain things about your injuries in a way you could understand?**   - Never - Sometimes - Usually - Always - Not Applicable | **9. During your follow-up appointments or visits, how often did your healthcare providers explain things in a way you could understand?**   - Never - Sometimes - Usually - Always | **Item revised.**  We broadened the item by eliminating the reference to injury. Explanations by healthcare practitioners may go beyond the scope of just the injury and may concern recovery more broadly. |
| **13. During your follow-up appointments, when you expressed concerns or frustrations how often did your healthcare providers take action to deal with them?**   - Never - Sometimes - Usually - Always - Not Applicable |  | **Deleted.**  High “not applicable” response rate in the validation study (24%). |
| **14. After being discharged from the hospital, how often was the information you received from your different healthcare providers consistent?**   - Never - Sometimes - Usually - Always - Not Applicable |  | **Deleted.**  High “not applicable” response rate in the validation study (32%). |
| **15. Since being discharged from the hospital, how often have you experienced healthcare that was unsafe?**   - Never - Sometimes - Usually - Always - Not Applicable | **11. Since being discharged from the hospital, how often have you experienced healthcare that was unsafe?**   - Never - Sometimes - Usually - Always | **Item maintained.**  There was limited response variation however, it is a crucially important topic and this allows comparisons to perceived safety of care in-hospital. |
| **16. In your opinion, how much information about your hospital stay was communicated to your family physician or general practitioner on a scale of zero to ten, zero being no information and ten being all the information?**   - **0** No Information - **1** - **2** - **3** - **4** - **5** - **6** - **7** - **8** - **9** - **10** All the Information - Not able to answer | **10. Did your family physician or general practitioner receive information from the hospital about your injuries, your hospital stay, or the care you would need to continue your recovery?**   - No - Yes, but they wanted more information - Yes and they got all the information they wanted - I don’t know - I haven’t seen a family physician or general practitioner since being discharged | **Item revised. Responses revised.**  Some patients had difficulty answering this item in the validation study. Many suggested they did not know. Some participants suggested their GPs knew of their hospital stay but didn’t know the details of their injuries. Other participants reported that their GPs were well-informed about their injuries and recovery plan. This item was a strong predictor of a patient’s overall rating of the quality of care so we elected to keep and revise the item. We have incorporated more description about the types of information the GP may have received and provided response options that will allow all participants to answer. The options eliminate the need for a screener item. |
| **17. How well were you guided through the recovery process by your healthcare providers after being discharged from the hospital on a scale of zero to ten, zero being poor guidance, ten being excellent guidance.**   - **0** Poor Guidance - **1** - **2** - **3** - **4** - **5** - **6** - **7** - **8** - **9** - **10** Excellent Guidance | **12. On a scale of zero to ten, how well have you been guided through the recovery process by your healthcare providers since being discharged from the hospital, zero being poor guidance, ten being excellent guidance.**   - **0** Poor Guidance - **1** - **2** - **3** - **4** - **5** - **6** - **7** - **8** - **9** - **10** Excellent Guidance | **Item revised.**  Wording slightly revised for clarity. This revision was based on feedback provided by participants during telephone interviews. Also reworded to reflect the fact that many participants may have ongoing treatment and recovery when the measure is used. |
| **18. On a scale of zero to ten, please provide an overall rating of the follow-up care you have received so far since being discharged from the hospital, zero being the worst injury care possible, ten being the best injury care possible.**   - **0** Worst Injury Care Possible - **1** - **2** - **3** - **4** - **5** - **6** - **7** - **8** - **9** - **10** Best Injury Care Possible | **13.** **On a scale of zero to ten, please provide an overall rating of the follow-up care, appointments, visits, and information you have received so far since being discharged from the hospital, zero being the worst injury care possible, ten being the best injury care possible.**   - **0** Worst Injury Care Possible - **1** - **2** - **3** - **4** - **5** - **6** - **7** - **8** - **9** - **10** Best Injury Care Possible | **Item revised.**  During post-acute care interviews participants would report that they didn’t receive care even if they had reported attending appointments. Therefore, we changed the wording to cover all types of “follow-up care” a person may receive. |
| **19. Which of the following options best describes your overall health right now?**   - Excellent - Very Good - Good - Fair - Poor | **14. Which of the following options best describes your current overall physical health?**   - Excellent - Very good - Good - Fair - Poor   **15. Which of the following options best describes your current overall mental or emotional health?**   - Excellent - Very good - Good - Fair - Poor | **Item revised.**  Item split into two items. Revised to match the Canadian Institute of Health Information patient experience survey. Allows for assessment of physical and mental health. |
| **20. What was the best aspect of the follow-up care you have received so far since discharged from the hospital?**   - *Free text* |  | **Deleted.**  To reduce item count the measure will only include one free text item. Most patients duplicated comments between free text items. |
| **21. What was the worst aspect of the follow-up care you have received so far since being discharged from the hospital?**   - *Free text* |  | **Deleted.**  To reduce item count the measure will only include one free text item. Most patients duplicated comments between free text items. |
| **22. What can we do to improve care for patients after they are discharged from hospital?**   - *Free text* | **16. Please provide comments on how we can improve injury care for patients after they are discharged from hospital?** | **Item revised.** |
