# Supplementary material for: Short form version of the Quality of Trauma Care Patient-Reported Experience Measure (SF QTAC-PREM)
Source: BMC Res Notes. 2017 Dec 6;10:693. doi: 10.1186/s13104-017-3031-9 (PMC5718023; doi:10.1186/s13104-017-3031-9)
Supplement: Supplementary file 7 — Additional file 7. Short form acute care QTAC-PREM ordinal logistic regression results, Short form post-acute care QTAC-PREM ordinal logistic regression results. [file 13104_2017_3031_MOESM7_ESM.docx]

| Supplementary File 7a. Short form acute care QTAC-PREM ordinal logistic regression results^a^ | | | | | |
| --- | --- | --- | --- | --- | --- |
| **Subscales^b^** | Coefficient | Std. Error | Z | P>\|z\| | 95% Conf. Interval |
| **Information and communication** | 14.38 | 2.09 | 6.87 | <0.001 | 10.28 to 18.49 |
| **Clinical and ancillary care** | 3.94 | 0.94 | 4.19 | <0.001 | 2.10 to 5.78 |
| Cut 1 | 10.75 | 1.62 | . | . | 7.57 to 13.93 |
| Cut 2 | 14.50 | 1.94 | . | . |  |
| ^a^Sample size n=154; R^2^=0.47; Brant test for proportional odds p= 0.40 | | | | | |

| Supplementary File 7b. Short form post-acute care QTAC-PREM ordinal logistic regression results^a^ | | | | | |
| --- | --- | --- | --- | --- | --- |
| **Subscales** | Coefficient | Std. Error | Z | P>\|z\| | 95% Conf. Interval |
| **Post-discharge information and communication** | 11.41 | 1.94 | 5.87 | <0.001 | 7.60 to 15.22 |
| Item 2: Pain management | 0.29 | 0.17 | 1.69 | 0.09 | -0.05 to 0.63 |
| Item 5: Difficulty scheduling appointments^b^ | - | - | - | - | - |
| Item 10: Family physician informed | -0.19 | 0.08 | -2.46 | 0.02 | -0.34 to -0.04 |
| Item 11: Perceived safety of care | 0.42 | 0.75 | 0.57 | 0.57 | -1.04 to 1.88 |
| Cut 1 | 6.41 | 2.31 | . | . | 1.88 to 10.94 |
| Cut 2 | 9.80 | 2.44 | . | . | 5.01 to 14.59 |
| ^a^Sample size n=117; R^2^=0.29; Brant test p=0.91.  This item underwent major revision for inclusion on the short form and therefore could not be assessed using data from the long form validation study. It was not included in the regression model. | | | | | |
